# What a Transfusion Physician Should Know about Monkeypox Virus: Barriers to and Risks for Transmission, and Possible Mitigation Strategies

**DOI:** 10.3390/diagnostics12092200

**Published:** 2022-09-11

**Authors:** Daniele Focosi, Massimo Franchini

**Affiliations:** 1 North-Western Tuscany Blood Bank, Pisa University Hospital, 56124 Pisa, Italy; 2 Division of Transfusion Medicine, Carlo Poma Hospital, 46100 Mantua, Italy

**Keywords:** monkeypox, donation deferrals, MSM

## Abstract

The ongoing monkeypox pandemic is posing new challenges to the transfusion community. While to date most regulatory agencies recommend deferrals for cases and contacts, we summarize here arguments for introduction of universal PCR testing of MPXV in blood donations and donated tissue/organs.

Monkeypox (MPX), a human zoonosis known for decades to cause severe rash and encephalitis mostly in children and pregnant women in Western and Central Africa, has been rapidly emerging as a novel threat to blood safety around the globe since May 2022 [1]. MPX was declared a Public Health Emergency of International Concern (PHEIC) by the World Health Organization on 23 July 2022. As of 9 September 2022, the monkeypox virus (MPXV) pandemic has totaled more than 55,000 cases across the world (https://ourworldindata.org/monkeypox). 

Uncertainty remains regarding the main points to consider:

- **Transmissibility of MPXV with transfusion**. Whenever there is a viremic stage of infection, there is biological plausibility for transmission. The 2022 WHO MPX guidance reports that *“any viremia occurs early in the course of infection, usually in the prodromal period, and before skin lesions become manifest”* [2]. For human infections with MPXV, it is well known that from the initial site of infection the virus spreads via lymphatics causing low-level, primary viremia to reach the reticuloendothelial system (bone marrow, spleen and liver). Replication in these organs causes fever: from there a massive secondary, leukocyte-associated viremia leads to skin lesions [3]. Most of the recent MPXV cases in the USA showed localized lesions and 3/12 patients investigated were viremic as shown by PCR [4]. In both the cynomolgus [5] and the prairie dog MPXV respiratory challenge model, viral DNA was seen in blood on days 6–9 through day 17 post-infection [6]. The highest loads were seen on day 12, where viable virus was detected (2 × 10^6^ PFU/mL), while by day 24  viral DNA was undetectable [7]. Prevalence of viremia in symptomatic patients diagnosed during the 2022 outbreak ranges from 7% to 67%, with high median cycle thresholds (i.e., low viral loads) ranging from 34 to 36 (Table 1). Of interest, MPXV has not been cultured from blood yet, and viral load in whole blood does not differ from viral load in plasma (Prof. Fabrizio Maggi, personal communication), meaning no or very low cell-associated virus. Primary MPXV viremia in asymptomatic subjects (ideally traced contacts of positive cases) is likely to be higher, but it remains completely unstudied in humans. Studies of related human poxviruses are also useful for risk assessment. For example, cowpox virus DNA was detectable in whole blood, but not in serum as late as week four post-infection [8].
- **Prevalence of infectious donors in the overall blood donor population**. The majority of the worldwide population is fully susceptible to orthopoxviruses, since the smallpox vaccination campaign was terminated in 1980s worldwide [9]. While initially confined to the men who have sex with men (MSM) community, MPX cases are now increasingly recognized outside the MSM community, but around 98% of cases reported so far come from highly promiscuous members of the MSM community. In addition, previous surveys have shown that MSM are willing to donate [10] and likely not to comply with blood donation deferrals [11,12]. In 2012 the MSM population comprised approximately 7% of the U.S. male population [13] and represented an estimated 2.6% of male blood donors [14]. Blood donation policies governing MSM have significantly relaxed over time in USA, Canada, France, Denmark, Japan, The Netherlands, and UK, from an initial lifetime ban in the wake of the AIDS crisis to successive phases of time-based deferment requiring periods of sexual abstinence. In early April 2020, the Food and Drug Administration (FDA) in the USA shifted to a three-month deferral period after sex for MSM (including those in monogamous relationships) in response to urgent needs in the blood supply amid the COVID-19 pandemic [15].
- **Opportunities to intercept an infectious donor at the medical selection visit**. The classical exanthem is extremely likely to self-prevent donation because of social stigma and severe symptoms, but retrospective screening of MSM cohorts in the 2022 wave has shown that totally asymptomatic MPX clinical presentations are increasingly recognized even in HIV-positive subjects [16,17]. Half of the MPX cases do not have any concomitant infectious disease for which mandatory screening would prevent clinical usage of the donated unit.
- **Opportunities to intercept asymptomatic cases**. About 50% of cases so far were also HIV-positive (Table 1), meaning that mandatory HIV-1/2 nucleic acid testing and serology would intercept eventual blood donations.

Other points to consider are instead well consolidated:

- **Documented cases.** Transmission of human orthopoxviruses after blood transfusion within the medical literature are currently limited to only once with smallpox [18].
- **Inactivation during plasma fractionation**. Poxviruses have been considered the “worst case” for solvent-detergent virus inactivation steps [19,20], but the safety of plasma derivatives is nevertheless preserved. For vaccinia virus, a model closely resembling MPXV, fast inactivation to the assay detection limit, i.e., reduction of infectivity greater than 4 log_10_ within 10–20 min, was achieved with Triton X-100 or Nereid [21].

**Table 1:** MPXV DNA prevalence (%) and median cycle thresholds (Ct) in registry studies during the 2022 outbreak.

Country	HIV+ (%)	MPXV in plasma	Ref.
16 countries	218/528 (41%)	35/528 (7%) Median Ct n.a.	[22]
France	112/236 (29%)	8/26 (31%) Median Ct 36	[23]
Italy	15/36 (42%)	24/36 (67%) Median Ct 34	[24]

**Risk Mitigation Approaches**

Thus far, regulatory authorities have reacted in variegate manners [25] that largely ignore the possibility of asymptomatic or pauci-symptomatic MPXV carriers, and imply that contact tracing is 100% effective. 

Many regulatory authorities have rather focused on risks from MPX vaccinations. Viremia in VACV vaccinees seems to be an extremely rare event and seems to be detectable by PCR only between days three and seven after vaccination [26]. However, vaccination adverse effects can be linked to viremia in many cases [27]. The risks from eventual vaccina virus transmission are already minimal [26,28,29] and under full control with the current deferral guidelines [30].

In sight of the high risk of morbidity and mortality from MPXV, especially in children and pregnant women, and the restriction of the vast majority of MPX cases to the MSM community, I feel it is appropriate and urgent to act in order to further mitigate the risk of transmission. While focusing on a subgroup comes with the risk of raising stigma and requires voluntary disclosure, this should not discourage authorities from introducing additional public health measures. We evaluate here several possible risk mitigation approaches in MSM donors:

- Universal leukoreduction of blood components (i.e., removal of white blood cells by filtration) is unlikely to be useful, given the occurrence of MPXV in plasma.
- Reintroduction of donation deferral for unvaccinated MSM (i.e., those born after discontinuation of smallpox vaccination and who have not yet received novel MPX vaccines). This approach is accompanied by severe uncertainties about the duration of smallpox-vaccine-induced protection [31], and the occurrence of vaccine breakthrough infections (around 4% and mildly symptomatic [31] but with no data about viremia so far) with the novel MPX vaccines. Even if reassuring evidence will accumulate, this approach is likely to severely impact blood sufficiency.
- Serological disease screening for MPXV suffers from false negatives during the window period of infection, suffers from cross-reactivity with other poxviruses, and is currently available only at selected academic hospitals. Hence it is neither useful nor feasible.
- Usage of nucleic acid testing (NAT) [32]. Diagnostic MPXV cycle threshold (Ct) values ≥ 33 predict poorly or non-infectious specimens [33]. As shown in Table 1, a least some subjects have Ct < 33 at symptomatic stages, which suggests likely higher viral loads during the asymptomatic stages. Of course, caution would lead to donation discard in case of positive NAT, regardless of Ct values.
- Pathogen reduction technologies (PRT). Amotosalen/UVA inactivation reduces IHD-W strain of vaccinia virus by > 5.2 logs [34], and is hence expected to be effective against MPXV, too. No published data are available for alternative PRT platforms at this time.

Among the possible solutions, the most cost-effective is likely NAT testing, which should remain so even if the pandemic expands to categories other than MSM.

## Abbreviations

MPXV: monkeypox virus; MPX: monkeypox; MSM: males having sex with males.
